# Biomedical Applications of Polyhydroxyalkanoate in Tissue Engineering

**DOI:** 10.3390/polym14112141

**Published:** 2022-05-24

**Authors:** Thiruchelvi Pulingam, Jimmy Nelson Appaturi, Thaigarajan Parumasivam, Azura Ahmad, Kumar Sudesh

**Affiliations:** 1 School of Biological Sciences, Universiti Sains Malaysia, Penang 11800, Malaysia; thiruchelvi@usm.my (T.P.); azura.ahmad@usm.my (A.A.); 2 School of Chemical Sciences, Universiti Sains Malaysia, Penang 11800, Malaysia; jimmynelson@usm.my; 3 School of Pharmaceutical Sciences, Universiti Sains Malaysia, Penang 11800, Malaysia; thaigarp@usm.my

**Keywords:** Polyhydroxyalkanoate (PHA), scaffold, tissue engineering, biomedical, biodegradable, biocompatible

## Abstract

Tissue engineering technology aids in the regeneration of new tissue to replace damaged or wounded tissue. Three-dimensional biodegradable and porous scaffolds are often utilized in this area to mimic the structure and function of the extracellular matrix. Scaffold material and design are significant areas of biomaterial research and the most favorable material for seeding of in vitro and in vivo cells. Polyhydroxyalkanoates (PHAs) are biopolyesters (thermoplastic) that are appropriate for this application due to their biodegradability, thermo-processability, enhanced biocompatibility, mechanical properties, non-toxicity, and environmental origin. Additionally, they offer enormous potential for modification through biological, chemical and physical alteration, including blending with various other materials. PHAs are produced by bacterial fermentation under nutrient-limiting circumstances and have been reported to offer new perspectives for devices in biological applications. The present review discusses PHAs in the applications of conventional medical devices, especially for soft tissue (sutures, wound dressings, cardiac patches and blood vessels) and hard tissue (bone and cartilage scaffolds) regeneration applications. The paper also addresses a recent advance highlighting the usage of PHAs in implantable devices, such as heart valves, stents, nerve guidance conduits and nanoparticles, including drug delivery. This review summarizes the in vivo and in vitro biodegradability of PHAs and conducts an overview of current scientific research and achievements in the development of PHAs in the biomedical sector. In the future, PHAs may replace synthetic plastics as the material of choice for medical researchers and practitioners.

## 1. Introduction

**Figure 1:**
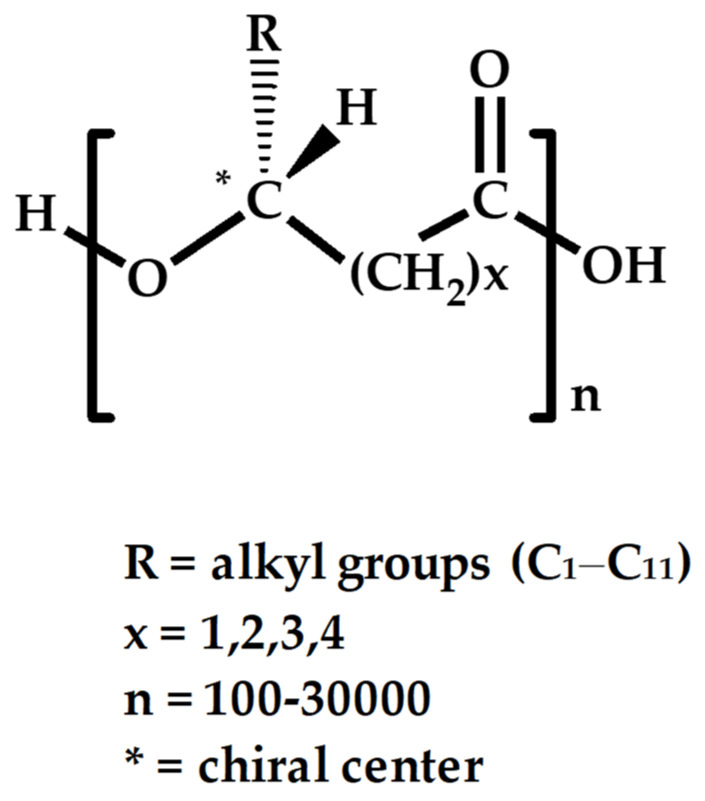
A generic chemical structure of polyhydroxyalkanoate biopolymer.

Natural biodegradable polymers, known as polyhydroxyalkanoates (PHAs), are created by aerobic and anaerobic bacteria, as well as certain extremophiles, under adverse circumstances [1,2]. PHAs are a diverse class of biomaterials composed of various hydroxyalkanoic acids (Figure 1), which are both biocompatible and biodegradable [3,4,5]. PHAs are bacterially produced straight polyesters that have been developed and tested as metabolic particles that accumulate in prokaryotic cell cytoplasm, among numerous other biopolymers [6]. As energy and carbon storage molecules, PHAs are accumulated by many microbes, such as *Bacillus*, *Pseudomonas*, *Aeromonas*, and aerobic, anaerobic, and photosynthetic bacteria, as well as archaea, when there is an excess carbon supply and nutrient depletion in the environment (e.g., nitrogen, phosphorus, sulfur or oxygen) [7,8,9]. Certain bacteria, such as *Alcaligenes latus* strains IAM 12664 T, have been demonstrated to be able to manufacture PHA without any nutritional limitations. [10]. At the moment, the structure and properties of PHA components produced via fermentation of bacteria differ significantly. This is primarily determined by microbial species, biosynthetic circumstances, and the carbon supply substrate used in the fermentation process, which alter the composition of PHA at the hydroxyalkanoate (HA) monomer level [2].

PHA is regularly categorized into two categories: short chain length (scl-PHAs) with four to five carbons, such as poly(3-hydroxyvalerate), (PHV) and poly(3-hydroxybutyrate), (PHB) and medium chain length (mcl-PHAs) with six to thirteen carbons, including poly(3-hydroxyhexanoates), (PHHx), and poly(3-hydroxyoctanoates) (PHO) [11,12]. Mcl-PHAs and scl-PHAs may be categorized as homo-polymers (scl-PHAs and/or mcl-PHAs) or copolymers (a combination of various monomers of scl-PHA and/or mcl-PHA monomers) based on the change in the length of the side chain caused by the addition of carbon atoms [13]. Due to its high melting point, low ductility, and high crystallinity, PHB is an example of a scl-PHA with outstanding biomaterial properties for hard tissue engineering [11]. In comparison, due to its elastomeric characteristics and low melting-point, mcl-PHA is an excellent scaffolding material for the majority of soft tissue engineering applications [11,14]. Covalently connected copolymers may be created by adjusting the substrate sources throughout the manufacturing process, which can affect the mechanical and thermal characteristics of the PHAs [15].

The thermal and mechanical characteristics of several PHA family members, such as scl-PHA, are comparable to those of petroleum-based polymers, such as polypropylene [16]. Biodegradation of PHA has also been demonstrated to be facilitated by a wide range of microorganisms in various environments. In vivo, some PHAs can be degraded by lipases and esterases [17,18]. For example, 3-hydroxybutyrate (3HB), the most well-studied of all PHAs, was initially found in 1926 in the bacterium *Bacillus megaterium* and other similar oligomers are found in bloodstreams of people and animals, indicating PHAs that are extremely biocompatible [19,20]. These polymers were created as biodegradable and renewable alternatives to petroleum-based plastics [3]. PHBV and PHB were more widely available as a result of this commercial interest, allowing for their assessment as medicinal biomaterials, such as in tissue engineering and drug delivery applications [21].

It has been reported that mcl-PHA (elastic) with 6 to 14 carbons has been used for soft tissue implants in a number of different applications [22]. However, PHAs have various limitations, including a lack of mechanical stability, a slow biodegradation rate, and crystalline nature, to name a few examples. [2]. Due to this, attempts have been undertaken to create enhanced PHA manufacturing procedures, which usually include the use of genetic manipulation and synthetic biological techniques, among other approaches [23]. As a result of these efforts, novel composite materials containing PHA in conjunction with other suitable organic or inorganic components have been developed. When this occurs, the resultant products are blends and composites with differing compositions, which have the potential to overcome the disadvantages of plain PHA [24,25]. Hence, they have the potential to be utilized as promising agents in a wide range of pharmaceutical, surgical, and therapeutic applications, due to their great potency and utility [26,27].

**Figure 2:**
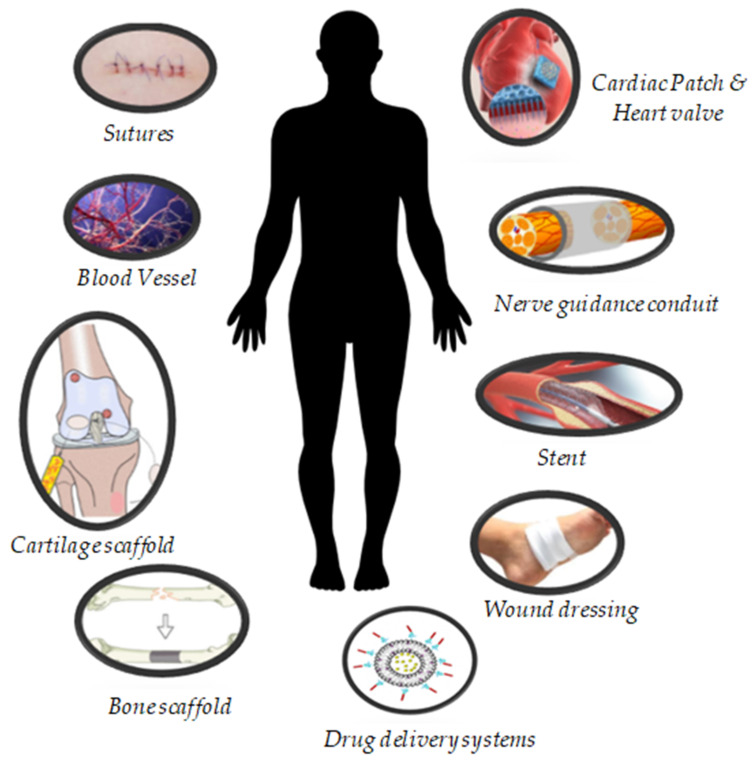
Applications and development of PHAs for the biomedical sector, especially in the tissue engineering field.

Thus, the achievements of PHA in tissue regeneration over the last six years are examined and reviewed in this paper, as well as presentation of a viewpoint on PHA’s biomedical application trends. Figure 2 depicts a conceptual model of the evolution of PHAs with substantial applications in the healthcare field. Finally, the degradation activity of PHA polymers is discussed to further comprehend the characteristics of this biomaterial when used in the medical sector. The advancement of PHAs in individualized, targeted tissue repair and regeneration has been aided mainly by the medical-engineering multidisciplinary nexus, which has also provided a new paradigm for advancement in the field of biomedical engineering.

## 2. Common types of PHA Used in Tissue Repair and Engineering

In the last decade, PHAs have drawn substantial interest for tissue engineering and as biomaterials for scaffolds, due to their thermal and mechanical properties, as well as biocompatibility and biodegradability, and feasibility to blend with other polymers to form copolymers [28,29,30,31,32]. The U.S. Food and Drug Administration (FDA) has approved PHAs for several biomedical applications [28,31]. Hence, the polymer has been widely investigated for artificial cartilages, dressing of wounds, artificial vessels, tendon healing, tubular substitutes, heart valves, bone grafts and stents for nerve repair [28,29,30,31,32,33,34], as summarized in Table 1. In fact, up to date, the US FDA has approved a number of PHA-based products, including TephaFLEX^®^ (sutures made of poly (4-hydroxybutyrate), P(4HB)) [31,35], Phasix^TM^ (mesh made from P(4HB) for the treatment of hernia) [31,36] and Monomax^®^ (sutures made of P(4HB)) [31,37].

Among the various PHAs, PHB and its copolymers, including PHBV copolymer and PHV homopolymer, are widely employed in biomedical tissue engineering owing to their superior biocompatibility compared to other degradable polymers [29]. These polymers have been reported to not trigger an adverse immune response, such as inflammatory response, calcification, and malignization when implanted in vivo [67]. The reported PHA films also did not cause necrosis, abscess or tumorigenesis up to six weeks upon subcutaneous implantation in rats [68]. In contrast, these PHA films have been claimed to support the growth of various cells (i.e., mesenchymal stem cell, epithelial, chondrocyte and fibroblast cells) as well as promote cell adhesion and proliferation [34,69]. At the same time, good hemocompatibility with minimal recruitment of lymphocytes was observed when these polymers were in contact with blood [70]. These findings reveal the excellent biocompatibility of PHAs as a biomaterial for tissue engineering. This is because the monomers in these polymers are natural metabolites in the human system that may be used by the body and then expelled as carbon dioxide when no longer needed [33]. For instance, the monomer, 3-hydroxybutyric acids (3HB) of PHB is a metabolite from fatty acids oxidation in humans [71,72]. Similarly, in vivo degradation of PHBV produces 3-hydroxybutyrate, a major ketone in the blood [73].

Conversely, PHB suffers from high crystallinity and brittleness, requires relatively longer duration for degradation and is also hydrophobic in nature with low thermal stability, which could limit its application for tissue engineering [74,75]. To overcome these barriers, PHAs can be physically or chemically blended with other polymers to increase ductility and flexibility. For instance, electrospinning of PHBHHx and poly (L-lactic acid) (PLLA) mixture produced fibrous membranes with higher elongation and lower tensile modulus as compared to the cast membranes [76]. PLLA has also been shown to degrade faster than PHBHHx [76]. Similarly, the crystallinity of PHBV was reported to decrease when blended with PLLA in a scaffold containing bioactive hydroxyapatite using emulsion/freeze-drying techniques [77]. This is expected to enhance the degradation rate of the matrix [77].

Another strategy is copolymerization via bacterial fermentation with different precursors to produce PHAs with high flexibility and ductility. Most bacteria produce a copolymer in the presence of suitable precursors. For instance, PHBV is a copolymer with less crystallinity, greater elasticity and greater strength than PHB, which can be produced by adding valerate in the fermentation medium [78]. However, the high production costs and low productivity rate of this approach often restrain the widespread usage of PHBV for tissue engineering applications [75,79]. Various strategies have been reported to overcome these barriers, including (i) usage of wild species bacteria that can readily produce PHBV without the addition of any precursors [80,81,82] and (ii) utilization of organic waste substrates for efficient production with low production costs [83,84]. Policastro et al. (2021) have extensively reviewed various PHBV production strategies to enhance polymer productivity with reduced cost based on techno-economic analysis [85].

## 3. Biomedical Applications of PHA

PHA has gained great interest in the area of biological tissue healing and engineering because of its structural variety and excellent physicochemical properties. PHA has been employed in soft and hard tissue regeneration fields, which include nerve repair, regeneration of heart valves and organs, and vascular, bone and cartilage tissue engineering [11,33]. PHA scaffolds are also employed to transport cells to a specific site during insertion, wherein cells are initially seeded onto the PHA-based scaffolding prior to utilization. Mcl-PHAs are most employed in soft tissue applications due to their elastic characteristics and are commonly used for sutures, wound dressings, cardiac patches, heart valves, cartilage tissue and nerve conduits. The capacity to change the mechanical qualities of PHAs via the production of blends or copolymers, making the composites substantially stiffer for uses such as cartilage tissue, has demonstrated intriguing results [86,87]. On the other hand, PHB, a scl-PHA, is employed in hard tissue applications, such as bone implants, because it offers the necessary mechanical rigidity required to create feasible degradable scaffolds with adequate mechanical and physical qualities. A wide range of PHA composites and blends have been produced in this field [88,89]. The following sections review a wide range of biomedical devices based on the use of PHAs, as well as their many uses.

### 3.1. Soft Tissue Engineering

#### 3.1.1. Sutures

Skin may regenerate on its own and, in rare situations, small skin injuries can cure themselves [11,90]. However, depending on the length and severity of the defect, the degree of microbial invasion, and the patient’s health, skin transplants and tissue engineered skin may be necessary in the event of severe defects, as well as material intervention. such as sutures and dressings of wounds [91]. Sutures are divided into two categories: absorbable and nonabsorbable. They are utilized for a variety of purposes, including ligation, tissue attachment, hemostasis, and wound repair [92]. Normally, the tensile strength of absorbable sutures degrades after two months of implantation and can be degraded into non-toxic compounds [93]. Therefore, the most desired qualities of absorbable sutures are biocompatibility, ease of tying and gripping, an even surface texture, high tensile strength, susceptibility to bacterial growth, ease of sterilization, and ultimate absorption and elimination [94].

Natural and synthetic absorbable sutures are available on the market, including natural catgut and synthetic polydioxanone [PDS], polyglyconate, polygalactin-910 [Vicryl^®^], polygalactin-910 rapide [Vicryl Rapide^®^], and polyglecaprone-25 [Monocryl]. In order to prevent the spread of Creutzfeldt-Jakob disease (CJD), the use of natural sutures has been restricted [3]. As a result, in the mid-1960s, PHAs were discovered to be an excellent material for usage as sutures since they met the anticipated qualities of absorbable sutures [95]. Sutures made of PHB and PHBV have been shown to give the strength needed for myofascial wound healing [67]. These sutures have been tested in comparison to other naturally absorbable (catgut) and non–absorbable (silk) sutures to determine their effectiveness. Wistar rats implanted with PHBV and PHB sutures demonstrated the typical extended (during the post-surgery monitoring period) and pronounced macrophagal stage. An in vivo investigation by Shishatskaya and colleagues showed no evidence of immediate vascular reactivity or of any undesirable consequences, such as suppurative infection, necrosis, fibrous capsule calcification, or carcinogenesis, for one year at the implantation site [96]. The tissue response to the insertion of PHBV and PHB sutures is consistent with the standard model of wound healing and the response to a foreign object invasion. Furthermore, no variations in the full set of physiological biochemical indicators, blood structure, animal sizes and major organ weights, biochemistry, and lymphatic tissue response were seen between the test and the control [96].

P(4HB) homopolymer has been discovered to be a promising material for absorbable sutures because its breakdown products are far less acidic than poly(glycolic acid) (PGA) or polycaprolactone (PCL), and it breaks down faster than PCL, PLLA and other PHAs, such as PHB [97]. By 2007, two other PHA-based suture materials–Phantom Fiber^TM^ (Tornier Co., Bloomington, ID, USA) and MonoMax^®^ (Braun Surgical Co., Hessen, Germany)—had been FDA-approved and were prepared using P(4HB) [91]. The current focus of PHA suture research is on further altering PHBHHx and PHBV for use as surgical sutures. Mixing the two low molecular weight polymers PLLA and PHBHHx (ratio 80:20) as a film boosted toughening, mechanical characteristics, and degradation rate, validating the blend as an ideal selection of materials to manufacture resorbable healthcare sutures [98].

**Figure 3:**
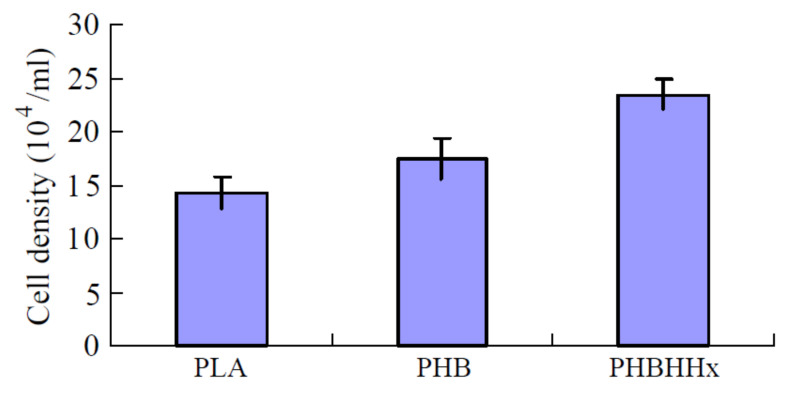
After 10 days of incubation, MTT assay for rabbit bone marrow cell proliferation on PLA, PHB, and PHBHHx scaffolds.

When compared to the poly(lactic acid) (PLA) and homopolymer PHB, the copolymer PHBHHx enhanced osteoblast adhesion and proliferation of rabbit bone marrow cells (Figure 3) [99]. Indeed, PHBHHx aided in the chondrogenesis of human BMSCs [100]. P(3HB-*co*-4HB-*co*-3HHx), a terpolymer, has shown even more potential for MSC differentiation than the copolymer PHBHHx [101]. MSCs were successfully grown on scaffolds made of PHBHHx and collagen [102]. Recent research found that the expression of several distinct integrins was discovered when P(3HBV-*co*-3HHx) was shown to have a significant influence on inducing osteoblast death. Various PHA films [PHB, PHBV, PHBHHx, and P(3HBV-*co*-3HHx)] were used to make it easier for these osteoblasts to adhere and proliferate. Surprisingly, the P(3HBV-co-3HHx) films exhibited less attachment, slowed cell proliferation, and increased apoptosis [103].

#### 3.1.2. Wound Dressing

One of the key difficulties facing health and clinical sectors is improving skin wound healing through the use of novel technologies and materials. Natural and synthetic polymers are receiving greater attention in research where importance has been placed on the properties of these materials to correspond to the kind of skin injury as well as the stage and progress of the healing activity. The wound dressing material must be non-adherent, simple to apply, non-toxic, sterile, and also antibacterial in order to be appropriate for the wound type [104]. Furthermore, the selected material must stimulate angiogenesis, retain a moist environment, enable exchange of gas and most importantly allow the growth and migration of fibroblast, keratinocyte and epidermal cells. Previous research conducted using PHAs have reported their great potential for use in wound dressing and have shown that fibroblast and keratinocyte cell types are able to adhere and grow more readily on PHA-based materials than on synthetic materials like PLLA [105].

**Figure 4:**
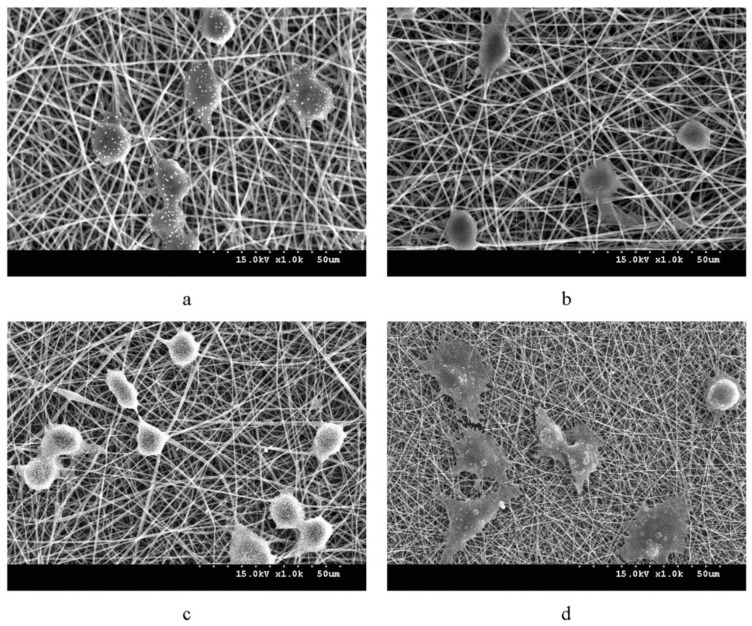
Adhesion of keratin, gelatin, and collagen to the PHBV polymer solution on the growth of fibroblasts has shown that collagen fibers with keratin fibers provided the best adhesion and proliferation reaction. (**a**) PHBV/keratin (**b**) PHBV/gelatin (**c**) and PHBV/collagen (**d**) mats. Reprinted from Wang et al. (2016) [108] with permission from Elsevier.

Vigneswari et al. (2016) reported that P34HB nanofibers/collagen peptides promoted the adhesion and proliferation of murine fibroblast cells (L929) and, furthermore, an in vivo investigation using these polymer nanofibers was shown to be substantially better at inducing the healing process (98%) than a control therapy using gauze (63%) [106]. Moreover, electro-spun PHBV mats treated with keratin have been shown to enhance regeneration and wound healing in studies [107]. Wang et al. (2016) investigated the effects of introducing gelatin, collagen and keratin to a PHBV polymer solution on fibroblast development, finding that collagen fibers combined with keratin fibers offered the greatest adherence and proliferation, as shown in Figure 4; although the collagen fibers with keratin fibers had satisfactory results as well [108]. Similarly, Shishatskaya et al. (2016) reported the use of P34HB based electro-spun fibers in wound healing contributed to high elasticity and low crystallinity values [109]. It was shown that patients with wounds treated with the cell-loaded P34HB membrane recovered 1.4 times faster than those who received the cell-free membrane and 3.5 times quicker than those who received the eschar membrane (control). The cell-loaded membrane group showed complete healing after 14 days, whereas the pure P34HB meshes and control groups showed about 90% and 70% area decrease, respectively [109]. As a result, the use of PHAs in wound-healing applications enhances important qualities, such as cell growth, which ultimately results in improved tissue repair.

#### 3.1.3. Cardiac Patch

PHAs have been studied for several parts of cardiac tissue engineering due to their outstanding biocompatibility and comprehensive mechanical features. For example, regenerative cardiac patches have been studied as whole scaffolds or as coatings to improve the functionalization and mechanical qualities of grafts of decellularized organ or other polymers. PHAs, namely PHB, have been studied for their applicability as anti-adhesive pericardial patches to be utilized during cardiac surgery to avoid adhesions or for artery augmentation in several studies [110,111,112]. Duvernoy et al. (1995) used the PHB cardiac patch in human patients for the first time and reported that computed tomography (CT) revealed a 27% decrease in adhesions between both the PHB patch and the heart surfaces compared to the non-patch group [110].

Bagdadi et al. (2018) produced a multifunctional PHO heart patch with mechanical strength comparable to myocardial tissue for cardiac tissue patches. When tested against newborn ventricular rat myocytes (NVRM), biocompatibility of the PHO patches demonstrated that the polymer was equivalent to collagen in response to cell survival, proliferation, and adhesion. Furthermore, no deleterious effects on adult cardiomyocyte contraction were seen when mature cardiomyocytes were seeded onto PHO patches [113]. Additionally, Castellano et al. (2014) linked PHB along with scaffolds of PLA, PCL and polyamide (PA) in infarcted rat hearts for cardiac repair. Although both PHB and PCL materials were efficient in minimizing negative ventricular remodeling, only PHB was able to generate significant angiogenesis in the heart tissue [114]. These results suggest that particular PHAs possess ideal material qualities for cardiovascular uses and the ability to sustain heart muscle stresses, providing PHA-based patches with a major competitive edge.

#### 3.1.4. Blood Vessel

Globally, blood vessel illnesses have resulted in an increase in fatality. Therefore, tissue engineering (TE) is becoming a viable therapy option for blood vessel disorders. Functional TE blood vessels combining living cells with polymeric scaffolds might be an effective alternative for dysfunctional blood vessels in therapeutic applications [115]. Chemically produced polymers, in conjunction with natural macromolecules, have been extensively researched as scaffolds for TE applications. A good blood vascular scaffold must comply with the following requirements: (1) outstanding mechanical characteristics and degradability after complete development of blood vessel generating cells; (2) ability to encourage cell growth and remodeling; and (3) no stimulation of stenosis, thrombosis, calcification, or infection [115].

In a large animal model, Opitz et al. (2004) [116] employed P(4HB) as a scaffolding material to tissue design a whole section of the descending aorta. Despite a fairly short in vitro timeframe prior to implantation (2 weeks), all blood arteries were able to tolerate systemic blood pressure for up to 3 months. This excellent outcome is most likely due to the P(4HB) scaffolds’ high flexibility and the presence of previously organized tissue at the time of implantation. In a similar study by Opitz et al., [117] tissue engineered blood vessels (TEBV) were implanted in the aortas of adolescent lambs and proven to be fully functioning for up to 3 months. The graft was still functional after 6 months, although it was significantly dilated owing to insufficient elastic fiber production.

PHB and P34HB comprising 0–40 mol% of 4-hydroxybutyrate (4HB) have been explored for their promising applications as rabbit blood vessel smooth muscle cell (RaSMC) due to their tunable elasticity, flexibility, capacity to stimulate elastin production, and strength [2,115]. P(HB-20 mol%-4HB) films produced more elastin by about 160% and had a lengthier elastin production time than P(HB-12 mol%-HHx) films. These are a viable choice for the development of a material for synthetic blood vessels. Recently, a melt processing approach was used to make bionanocomposites from bacterial cellulose nanofibers (BC) and PHO [118]. The addition of only 3 wt% BC to PHO enhanced thermostability by 25 °C and strengthened it, increasing the Young’s modulus by 76% and the tensile strength by 44%. The biocompatibility study demonstrated that PHO/BC nanocomposite with 3 wt% BC had low pro-inflammatory immune reaction and increased cell adhesion, confirming the bionanocomposite’s high practicality for in vivo application of tissue created blood arteries [118].

### 3.2. Hard Tissue Engineering

#### 3.2.1. Bone Scaffold

Bone tissue engineering, as an alternative to bone transplants, entails the use of a suitable biocompatible scaffold alone or in cooperation with other cells or bioactive substances. These scaffolds should be comprised of biodegradable polymers that are porous and capable of mechanical support and bone regrowth [119,120]. Large bone deficiencies, such as those caused by resections of bone tumors and severe fractures, necessitate surgical intervention using allografts, xenografts, autografts, or biomaterials-based bone implants. PHAs have been extensively studied for bone repair due to their biodegradation rates, biocompatibility, and improved mechanical qualities. Ding et al. (2016) reported that osteoblast-like cells (MG-63s) on polyhydroxybutyrate/poly(-caprolactone)/58S Sol–Gel Bioactive Glass electro-spun hybrid scaffold were favorable for cell adhesion of MG-63 and also slightly increased cell viability. Additionally, the 58S bioactive glass sol comprising the hybrid scaffold substantially increased alkaline phosphatase activity (ALP) as well [121].

**Figure 5:**
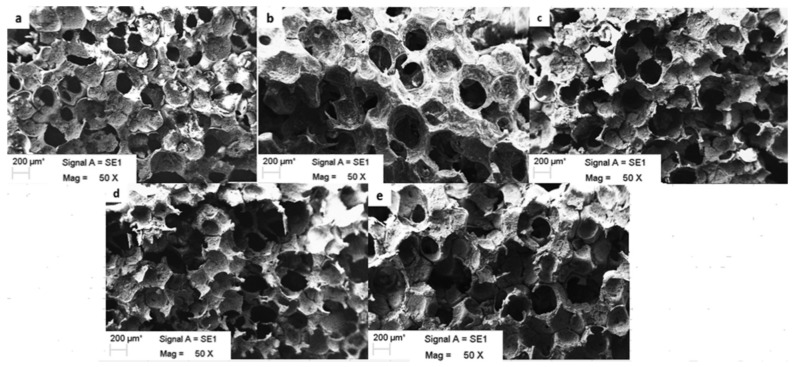
Scaffold with PHB-Chitosan (Cs)/MWCNTs bio-glass nanocomposite coating deposited on nano-bio-glass-titania and the PHB-Cs/1 wt% MWCNTs coated scaffold were found to improve the survival of MG-63 cells. (**a**) scaffold, uncoated (**b**) scaffold coated with PHB (**c**) scaffold coated with PHB-Cs (**d**) scaffold coated with PHB-Cs/0.5 wt% MWCNTs (**e**) scaffold coated with PHB-Cs/1 wt% MWCNTs. Reprinted from Parvizifard et al. [123] with permission from Elsevier.

PHBHHx is more biocompatible with smooth muscle cells, nerve cells, osteoblasts, fibroblasts, chondrocytes, and bone marrow mesenchymal stem cells (BMSCs) than PHB, PHBV, and PLA. Its degradation products, like oligo-(3-hydroxybutyrate), oligo-(3-hydroxybutyrate-co-3-hydroxyhexanoate) and 3HB, were found to be non-toxic as well [122]. Recently, Parvizifard et al. (2020) established PHB-Chitosan/ multi-walled carbon nanotubes (MWCNTs) scaffold with bio-glass nanocomposite coating which improved MG-63 cell survival and proliferation while also increasing the alkaline phosphatase secretion. The hydrophilicity of chitosan and surface roughness of MWCNTs, as shown in Figure 5, encourage cell proliferation and growth. Additionally, MWCNTs’ enhanced surface roughness also improved protein binding, which resulted in better cell growth and proliferation [123]. The addition of segments of soft polymers, such as hydroxyvalerate, hydroxyhexanoate, hydroxyoctanoate and hydroxyoctanoate or PEG, have been found to enhance the mechanical qualities of PHB which decreases the stiffness of the PHB matrix and inhibits crystallization of the backbone of the PHB polymer [124]. These favorable findings suggest that PHA composites can be used in hard tissue engineering because of their osteo-inductive capabilities and ability to promote angiogenesis.

#### 3.2.2. Cartilage Scaffold

Chronic pain and incapacity can result from cartilage loss due to age-related degeneration, trauma or developmental problems. Typically, cartilage tissues are avascular and incapable of regeneration. As a consequence, complete knee replacement surgery is usually necessary to relieve ache, discomfort and disability in patients. Recent developments in cartilage tissue engineering utilizing materials like PHAs, on the other hand, have shown effectiveness in treating early cartilage deterioration and consequently present alternatives to full joint replacement. Recently, Mohammadalizadeh et al. (2020) developed a nanocomposite fibrous scaffold consisting of MWCNTs distributed in PHB-chitosan and this composite showed better tensile strength (close to 3 times greater), increased hydrophilicity and a reduced degradation rate. Improved cell adhesion and cell growth were noted among cultured chondrocytes, where the mechanical properties of the cells were similar to human articular cartilage [125].

Ching et al. (2016) fabricated electro-spun nanofibers of PHB and PHO blends to resemble collagen fibers usually observed in articular cartilage. All the PHB/PHO nanofiber blends demonstrated high cell survival and type II collagen expression in human articular chondrocytes, leading to the conclusion that the PHB/PHO 1:0.25 blend fibers most closely represent articular cartilage [126]. Similarly, PHAs were combined together with collagen type I to create solvent-cast films and 3D printed objects and a cell viability assay, conducted using C-20/A4 chondrocyte cell line. showed that PHA-containing samples were not harmful and offered a “supportive environment for chondrocyte activity” [127]. Therefore, due to their mechanical qualities, capacity to induce cell activity and, most significantly, the creation of collagen, PHAs may closely match genuine cartilage, making them the next-generation material in this field.

### 3.3. Implantable Devices

#### 3.3.1. Heart Valve

Heart tissue-engineered aids in the regeneration of functioning valve replacements in living hearts by using biomaterials designed in the form of the heart valve, followed by cell seeding [128]. PHA polymers have recently yielded some of the most promising findings in heart valve construction as compared to PLLA [129], PCL [130] and PGA [131]. In comparison to mcl-PHA, synthetic absorbable polyesters were too rigid to work as elastic leaflets within a tri-leaflet valve [132,133]. In an in vivo investigation in lambs, leaflets substituted with porous and comparatively more flexible P(3HHx-*co*-3HO)-PGA mesh were shown to be more appropriate [134]. In the pulmonary region, a co-polyester of 3-hydroxyhexanoate and 3-hydroxyoctanoate [P(3HHx-*co*-3HO)] and autologous cells produced superior outcomes with little stenosis and no thrombus development in a lamb model and there was also little regurgitation for up to 17 weeks following implantation [131,135].

Hoerstrup et al. [133] produced a permeable scaffold material in the form of a tri-leaflet heart valve employing a PGA nonwoven mesh liquid coated with P(4HB) in order to enable more quick tissue remodeling in vivo. In this in vivo experiment, implant of the tissue-engineered heart valve in place of the adolescent sheep’s native pulmonary valve was shown to perform properly, and echocardiography of the transplanted valves indicated functioning movable leaflets without stenosis, thrombosis, or aneurysm. Only eight weeks after insertion, the composite scaffolds were discovered to have fully decomposed, and by twenty weeks, it had been transferred to a different tissue-designed heart valve that nearly resembled the original valve.

#### 3.3.2. Stent

PHAs are highly polymerized and have molecular weights in the millions of Daltons range [136]. PHAs are biocompatible, elastomeric, thermoplastic, insoluble in water, nontoxic, piezoelectric, and most importantly, biodegradable [9]. When compared to other polymeric materials, the outstanding mechanical qualities of PHAs, along with higher biodegradability and biocompatibility, make PHAs a preferred option for a range of medical applications, such as the production of biodegradable stents. The use of drug-eluting stents has lately transformed the area of interventional cardiology, which was formerly dominated by bare-metal stents (BMSs). One of the primary difficulties with the use of metal stents in cardiac purposes is the eventual restenosis that may develop as a consequence of excessive blood vessel wall growth. [137]. The gold-standard metallic stent used in medical settings, for example, is associated with the risk of restenosis, which happens when the artery narrows again around an implanted stent. To prevent this, the creation of a biodegradable stent—which opens a blocked artery and then deteriorates before restenosis occurs—is a significant advance in this sector. [11]. Thus, PHAs satisfy the criteria for drug-delivery coating on stents (i.e., drug compatibility, capacity to survive processing, sterilization, and preservation, customizable formulation, and drug-release qualities) in this field. [3].

**Figure 6:**
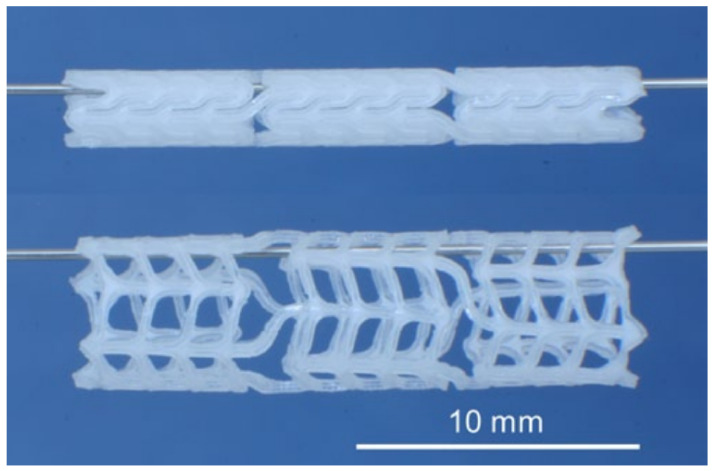
The biodegradable PLLA/P(4HB) stent unexpanded (**top**) and expanded (**bottom**). Reprinted from Kischkel et al. (2016) [138].

Biodegradable PLLA/P(4HB) stents (Figure 6) have recently been embedded in an porcine model and have demonstrated impressive outcomes, resulting in a decreased degree of stenosis when used in conjunction with an oral atorvastatin drug, when compared to the very same circumstances with fixed 316L stainless steel stents. [138]. Unverdorben et al. (2002) [139] investigated 11 polyhydroxybutyrate biodegradable stents and 13 stents (tantalum) which were fixed into the iliac arteries of white rabbits (New Zealand) for up to 210 days. PHB was reported to elicit severe inflammatory responses, such as a rise in collagen (2.4- to 8-fold versus native segments), thrombosis, and stent lumen shrinkage. The major cause of this is the intense inflammatory response elicited by the breakdown process, which involves considerable collagen and intercellular matrix formation and also monocyte buildup. Finally, clinical usage of the PHB stents was ruled out. Major improvements are necessary before they may be employed as stents, such as lowering the overall quantity of biopolymers, improving mechanics, and boosting radiopacity.

Markelova et al. (2008) [140] tested experimental PHA stent types for endobiliary prostheses based on their biological qualities. The experiments were carried out with the help of 20 adult mongrel dogs. The dogs were placed into three groups: negative control (intact dogs), positive control (dogs with implanted endobiliary silicon stents), and trial (dogs with PHA stents), and each group was observed for 100 days. They found no signs of inflammation at the end of the investigation, but cicatrical variations were assessed in the subhepatic spatium and free abdominal cavity. All of the PHA stents that had been implanted were still in their original locations. There was no inflammatory response or anastomosis after the trial. The liver and duodenum showed no macroscopic alterations. There were no pathological deviations in liver function. These encouraging findings support the conclusion of using PHA as endobiliary stents in bile passage reconstruction surgery.

#### 3.3.3. Nerve Guidance Conduit

Nerve injuries are widespread, and there is no simple formula for effective therapy. Entubulation techniques were developed as a result of better knowledge of the biological processes engaged in nerve regeneration and awareness that nerve grafts act as a guide for emerging neurons [141]. Nerve guidance conduits (NGCs) are entubulation implants that are used to preserve and aid in the regeneration of nerves after they have been damaged [11]. NGCs are typically hollow tubes that connect one end of the nerve to the other [142]. A scaffold for axonal proliferation, support cells such as stem cells or Schwann cells, growth factors, and an extracellular matrix are the fundamental components of nerve regeneration constructions [143].

Since NGCs are connected to a lack of extracellular matrix tissue, topographical cues, and cellular characteristics, the autograft approach is still used for significant injury gaps [144]. However, because autografts need second-site surgery, NGCs can be enhanced for critical gap injury application by including cellular treatments, surface modification, topographical enhancement, and physical guidance cues [145]. Furthermore, optimal NGCs for bridging nerve gaps must be biodegradable, freely accessible, quickly vascularized, antigenic, porous for oxygen passage, and prevent long-term compression. In 1999, Hazari et al. [146] and Ljungberg et al. [147] conducted up to a year’s worth of research on the usage of a nonwoven PHB sheet wrap to restore cats’ transected superficial radial nerves. Axonal regrowth was equal to closure with an epineural suture for a nerve gap of 2–3 mm, and the inflammatory response was normal. In a second investigation by Hazari et al. (1999) [148], the same material was employed to bridge a 10 mm intrinsic gap in rat sciatic nerves, and the outcomes were linked to an autograft transplant. After one month, the rate and volume of regeneration in the PHB conduit did not equal that of the autologous nerve transplant, but it did show excellent axonal regeneration and a low degree of inflammatory infiltration.

Taylor et al. (2020) [149] produced films by solvent casting techniques consisting of PHO/PHB 25:75 and 50:50 which significantly supported NG108-15 neuronal cell adhesion, proliferation, and differentiation compared to a PHO/PHB 75:25 blend and PHO films. Another research combined PHB and PHO to create blend fibers for regulating neuron cell development and differentiation in a directed manner [150]. A 75:25 PHB/PHO blend was utilized to fabricate electro-spun fibers as resorbable scaffolds to be used as internal guide lumen constructions in nerve conduits. For small, medium, and large diameter fibers, the obtained diameters were 2.4 µm, 3.7 µm, and 13.5 µm, respectively. Increased NG108-15 neuronal cell adhesion and differentiation were significantly supported by large fibers among the generated fibers [150].

In a one-pot bio-fabricated technique, a reduced graphene oxide (rGO) scaffold was decorated with gold nanoparticles and inserted into poly(3-hydroxybutyrate-*co*-12 mol% hydroxyhexanoate), P(3HB-*co*-12 mol %-3HHx) fibre [151]. The FESEM images revealed a porous mat-shaped matrix assembly with a fibrous hybrid character. Electrically conductive materials were investigated using the produced fibers. In vitro testing with Schwann cells demonstrated that all three scaffolds promoted Schwann cell adhesion, proliferation and migration. The research found that the addition of rGO/Au to the PHA scaffolds, as well as the application of electrical stimulation, enhanced these processes even further [151].

### 3.4. Drug Delivery Systems

#### Nanoparticles

In the 1960s, the United States developed a novel process known as drug delivery systems (DDSs), which utilized polymers with continual drug discharge. However, the potential application of PHA polymers was observed in the early 1990s [2]. PHA was employed in the DDS owing to its biocompatibility, biodegradability, and thermos processability, all of which are tolerated by the human body. Furthermore, PHA possesses a wide range of chemical compositions and functional groups, which allow for further chemical modification for application in drug delivery systems [152]. Modified PHA may be utilized to deliver the desired treatment for precise time frames, as well as to approach and identify specific regions of the body. The adjustable biodegradability of PHAs is mostly beneficial in the manufacture of devices that utilize patches, films, microparticles, nanoparticles, and prototypes [11,28].

PHB and PHBV have recently been actively researched for the development of effective medication delivery systems because they have favorable physicochemical properties, the capacity to connect with other polymers and, most significantly, no influence on platelet reactivity [2]. Rifampicin, sulbactam-cefoperazone, gentamicin, tetracycline, rubomycin, sulperazone, and rhodamine B isothiocyanate (RBITC) have all been studied using PHBV and PHB to evaluate drug delivery [153,154,155,156,157]. These polymers have been investigated for use as subcutaneous implants, compacted tablets for oral administration, and microparticulate transporters for intravenous administration [158]. Despite being used as a medium for controlled drug delivery by surface erosion, these polymers’ melting points and crystalline nature interfere with the drug release profile. PHB is mixed or copolymerized with other monomers to make polymers that are soft, stiffer, and have a lower melting point to overcome these drawbacks [2].

An injectable thermo-gelling PHB-based polymer was developed to decrease the deleterious effects of drugs on healthy cells. The PEG-PPG-PHB triblock polymer has thermo-gelling characteristics, is biocompatible, and has shown sustained release of doxorubicin and Paclitaxel. Intra-tumoral injection of these PTX-thermo-gels led to greater tumor mass decrease than the free medication or the thermo-gel alone [159]. A transesterification procedure was carried out by Zhou et al. (2012) [160] to create the mPEG-PHBV copolymer. These nanoparticles have been shown to be compatible, having the capacity to store and continuously deliver hydrophobic medications. A polymer composed of hyperbranched PEI and PHBV has been studied as a non-viral siRNA vector. In addition to improved transfection efficiency, this polymer displayed lower toxicity than bPEI against cell lines, while in vitro luciferase silencing was shown to be comparable to Lipofectamine 2000.

PHAs may be mixed with other substances to form composite molecules capable of transporting drugs into the bloodstream. The anti-inflammatory medication diclofenac may be delivered into the blood using PHA compound scaffolds of tricalcium phosphate (TCP) and PHO, that can be used to reduce inflammatory effects after invasive bone surgery [161]. The scaffolds had excellent biocompatibility with MC3T3-E1 mouse pre-osteoblast cells, and adding PHO to TCP scaffolds improved the scaffold’s compressive strength, which is necessary for bone healing, as well as resulting in longer diclofenac release [161]. Drug delivery strategies may also be tested using fibers. PLLA-PHB fibers containing dipyridamole were produced by non-solvent melt electrospinning and had a coarse texture and irregular size. However, PHB inclusion decreased PLLA crystalline nature. In vitro release investigations revealed that drug diffusion from the polymer (associated with fiber crystallinity) influenced the majority of the fiber release rate, although breakdown of the polymers’ ester groups also had an effect on it due to polymer degradation [162].

## 4. Biodegradability of PHA Used in the Medical Sector

### 4.1. In Vivo and In Vitro Biodegradation of PHA

**Figure 7:**
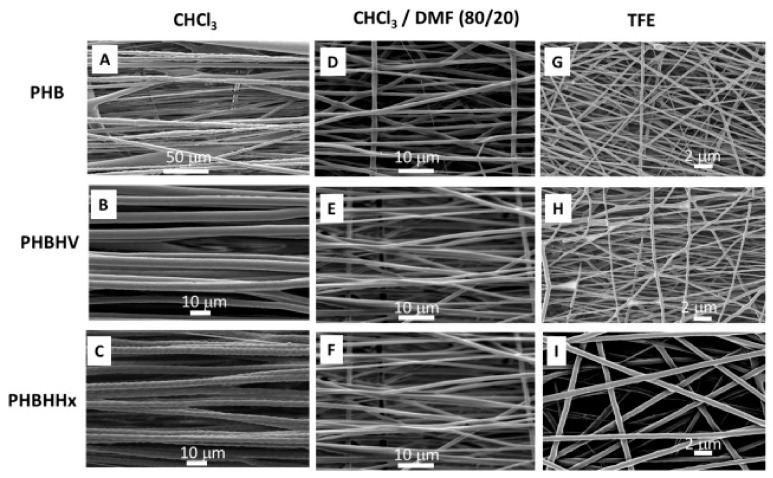
Scanning electron micrographs of electro-spun fibers of PHB 14% (wt/vol) in chloroform (CHCl_3_) (**A**), PHBV 30% (wt/vol) in CHCl_3_ (**B**), PHBHHx 10% (wt/vol) in CHCl_3_ (**C**), PHB 14% (wt/vol) in CHCl_3_/dimethylformamide (DMF) (**D**), PHBV 25% (wt/vol) in CHCl_3_/DMF (**E**), PHBHHx 10% (wt/vol) in CHCl_3_/DMF (**F**), PHB 10% (wt/vol) in trifluoroethanol (TFE) (**G**), PHBV 15% (wt/vol) in TFE (**H**), PHBHHx 10% (wt/vol) in TFE (**I**). Reprinted from Grande et al. (2017) [166] with permission from Elsevier.

PHA breakdown is governed by two major factors: environmental conditions and the characteristics of the PHA components. The degradation rate varies in different environments, such as water, soil, and physiological conditions [163], while the material properties of PHAs are mainly determined by monomer structure and compositions. PHA degradation in vitro has been studied extensively. However, because of the complexity of physiological fluids, in vitro results are not necessarily accurate predictors of in vivo behavior [164]. The degradation of PHA within human cells differs depending on tissues and the distribution method used to construct its various features, such as PHA thin films, scaffolds or nanofibers [165]. Grande et al. (2017) documented the surface morphology of numerous PHA-based electro-spun scaffolds created for biomedical applications, which are displayed in Figure 7 [166].

It has been shown that PHB, and its copolymers, are biodegraded in vivo primarily through the phagocytic capabilities of specialized cells (macrophages), as well as foreign body giant cells (FBGCs) and osteoclasts [167]. The implanted polymeric substance concurrently activates macrophages, enhancing their phagocytic activity [168,169]. The adherence of macrophages to the polymeric material’s surface is crucial. It was discovered that biodegradation of polymeric membranes occurs only when macrophages had adhered to their surfaces. If macrophages are unable to attach to the membrane, polymer breakdown does not proceed [170]. After 180 days of in vivo degradation in rats, Shishatskaya and coworkers (2005) [171] discovered local defects on the surface of some aligned fibers produced from PHB and PHBV copolymer. The flaws may have occurred as a consequence of polynuclear macrophages’ lysosomal activity, which accounted for 20–30% of the total number of cells in the silicon perforated diffusion chambers implanted subcutaneously in the rats’ neck folds [171].

Moreover, tissue enzymes have been shown to accelerate PHA degradation [172]. Freier and coworkers (2002) [173] implanted gastrointestinal patches made of a PHB/atactic PHB (at-PHB) in rat gastro-intestines and retrieved material fragments from only one out of the four test animasl after six weeks of implantation. The patch remnant was significantly degraded, with a molecular mass of just around 38% of its original value [173].

After implantation of PHB patches onto the stomach wall of rats, a rise in mRNA encoding pancreatic enzymes (a combination of enzymes comprising amylase, lipase, trypsin, a-chymotrypsin, and protease) was identified, which was linked to rapid drop in molecular weight. A similar result was reported by Zhuikov and coworkers (2021) [174]. Proliferation of 3T3 fibroblasts on PHB films was evaluated in vitro in phosphate buffered saline (PBS) at pH 7.4, 37 °C, and in PBS treated with 0.25 mg/mL pancreatic lipase, as well as against PLA and its 50:50 mix (PLA/PHB) films. The degradation rate for all polymers was found to be accelerated by pancreatic lipase [174].

With regards to PHA compositions, the incorporation of blends and other monomers alters the PHA material properties, reducing crystallinity, resulting in a drop in the melting point of the polymer, an increase in the polymer’s flexibility, and an increase in the polymer’s breakdown rate [173,175,176]. The degradation of PHA copolymers, which have low crystallinity, is more effectively degraded than homopolymer PHB, as shown in the decline in the *M*_n_ and *M*_w_ of the PHAs implanted subcutaneously to laboratory animals for a prolonged length of time [164,171,173,175]. Randomized chain scission in the amorphous areas of the polymer (e.g., 3-hydroxyhexanoate (3HHx), 3-hydroxyvalerate (3HV), etc.) was implicated in the faster degradation rates of PHA copolymers, causing an increase in water uptake that supports hydrolysis while leaving the crystalline regions temporarily intact. As a result, the polymer’s crystallinity increases during degradation followed by a decrease in overall crystallinity, whereas PHB hydrolysis was found in both amorphous and crystalline areas of the polymer matrix, where random chain scission was first seen. [164,173].

### 4.2. Biodegradability of PHA-Based Implants and Biocompatibility of Their In Vivo Degradation Products

Aside from sufficient mechanical properties and biocompatibility of PHA-based materials for medical purposes, degradation within clinically acceptable time frames is essential for use as a temporary implant material [173]. Due to their natural origins, PHAs are easily biodegraded, making them potentially valuable polymers in medical fields. PHAs are also suitable for medicinal applications, not only because of their low acidity and bioactivity, but also because of the non-toxicity of their biodegradation products [13].

Numerous studies have demonstrated the biocompatibility of PHA-based medical materials with cell tissue surrounding the implantation site [175,176,177]. The main concern after implantation is the fate of the PHA degradation products and whether the PHA residuals are biocompatible with the cells in places other than where they were intended. 3-hydroxybutyrate (3HB) and other PHA monomers, such as 4-hydroxybutyric acid (4HB) and 3-hydroxyhexanoate (3HHx), were shown to be compatible with cells in various parts of the body [164,177,180,181]. Additionally, they have a short half-life in vivo and human tolerance, making them ideal for cell and tissue growth [178]. 3HB is a natural component of blood, present in amounts ranging from 0.3 to 1.3 mM, and has been related to the formation of ketone bodies [179]. The presence of 3HB was shown to activate Ca^2+^ channels and enhance calcium influx in cultured cells, which supports the theory that monomers generated from PHA decomposition contribute to improving tissue regeneration [180]. 3HB and 4HB are less acidic than other biodegradable polyesters like PLA, PGA and poly (DL-lactide-co-glycolide) (PLGA) and may be removed from the human body within an hour. As degradation products, they may be found in nearly every part of the body [13].

Zhao et al. (2007) [181] found that PHBHHx, which consists of 3HB and 3HHx, enhanced the proliferation and differentiation of osteoblasts, leading to the hypothesis that 3HB is responsible for this phenomenon. The authors subsequently investigated the effect of 3HB on osteoblast growth in vitro and in anti-osteoporosis in vivo and reported an increased calcium deposition with increasing 3HB concentration from 0–0.1 g/L in both studies. PHA-based implants and their degradation products were also found to be degraded and fully resorbed in several in vivo studies [182,183]. In contrast, Volova et al. (2014) [184] noted noticeably slower bio-resorption of PHB in vivo; this might result in tissue inflammation at the implant site. Similarly, low in vivo degradation rates of PHA-based implants in the femora of living rats was observed in another study by Meischel et al. (2016) [185].

Despite long-term in vitro tissue response research, in vivo investigations in animal studies and clinical studies with people, the fate of PHA implants and the kinetics of the mechanisms leading to the loss of mechanical properties remains inconclusive. The disparities in the research findings may be attributed to the variability between sample properties, the use of different animal models, and the slow degradation of PHAs [186]. As evidenced by the work reviewed here, PHA-based medical materials, i.e., scaffolds, implants, sutures, etc., and their degradation products, are biocompatible, causing no inflammation and tissue response in cells, making them suitable for medical applications.

## 5. Conclusions

PHAs have gained popularity over the last two decades as a result of their versatile characteristics, biocompatibility, and bioresorbable attributes. This biomaterial has been used in a growing number of disciplines in recent years, including medicine, food packaging, ordinary chemicals, agriculture, and other fields. Additionally, they are ecologically favorable due to their sustainable development; nevertheless, more research is needed to produce larger PHA yields from waste materials in order to further improve sustainability. The biomedical industry is, without a question, the focus point for future PHA production and use. PHA and its derivatives have been widely used in therapeutic applications, such as cancer therapy, neurological and metabolic diseases, malnutrition, anti-diabetics, and environmental health, due to their economic practicality. PHA polymers have also lately been characterized as scaffolds (especially for cartilage tissue engineering), surgical sutures, transplants, and heart valves, drug delivery, cosmetic packaging, and films (in use for bone tissue engineering, post-surgery recuperation, neural regeneration, and stents). The growing significance of PHA polymers in the scientific world is defined by an increasing number of patents being registered. PHAs, in our opinion, are the future biomedical material for generating innovative scaffolds for tissue repair and regeneration, implants, and even prospective synthetic organ transplant difficulties. The use of computer-aided design (CAD) and additive fabrication techniques to create sophisticated 3D scaffolds for regenerative medicine applications is rapidly expanding. This has significant implications on PHA-based biomedical implants and regenerative medicine scaffolds. Additional study is needed, however, to simplify the use of PHAs in such technologies, since a variety of material property restrictions during production, such as material deterioration, would need to be managed. Lastly, regulatory process, improved production scale-up, and enhanced expense on PHAs other than P(4HB) and PHB will be necessary to realize the immense promise of this family of biocompatible and sustainable PHA polymers.

## Figures and Tables

**Table 1:** Application of different types of PHA in tissue engineering (published between years 2017 and 2022).

Type of PHA *	Therapeutic Agent	Combination with	Formulation Description	Technique	Key Finding	Ref
P34HB	-	-	Fibre scaffold	Electrospinning	The scaffold was interwoven with fibres and had good physical and chemical properties as well as induced cell adhesion and proliferation without cytotoxicity.	Fu, et al. [38]
P34HB	-	Poly(ethylene glycol)	Nanofiber membrane	Electrospinning	The nanofiber membrane supported cell adhesion, spreading, and proliferation and promoted osteoinduction capacity in vitro.	Wang, et al. [39]
P34HB	Actovegin or fibroblast cells	Bacterial cellulose	Film	Solvent evaporation	Bacterial cellulose and P3HB/4HB in combination with actovegin and fibroblast are more effective than a commercial wound dressing.	Volova, et al. [40]
PHB	-	Gelatin	Microfibers and nanofibers	Electrospinning	The combination fiber scaffolds were biocompatible and promoted fibroblast attachment and skin regeneration which makes it suitable for wound healing.	Sanhueza, et al. [41]
PHB	-	Carbon nanotubes	Nanotubes scaffolds	Electrospinning	The PHB and nanotubes composite caused mild foreign body type giant cell reaction, moderate vascularization, and mild inflammation.	Zarei, et al. [42]
PHB	-	Chitosan and nano-bioglass	Nanofiber scaffold	Electrospinning	The nanofiber scaffold showed significantly greater expression of dentin sialophosphoprotein, collagen type-I, and ALP making it suitable for dentin tissue engineering.	Khoroushi, et al. [43]
PHB	-	Polylactic acid	-	3D printing	The blending of polylactic acid and PHB can produce a stable tubular substitute for urethra replacement.	Findrik Balogová, et al. [44]
PHB	Bone marrow-derived mesenchymal stem cells	Chitosan	Conduit	Electrospinning	The conduit caused damage to the axons. The incorporation of chitosan with PHB resulted in a stronger and biodegradable nerve conduit.	Ozer, et al. [45]
PHB	Primary Schwann cells (SCs) or SC-like differentiated adipose-derived stem cells	-	Strips	-	PHB strip seeded with cells provides less muscle atrophy and greater axon myelination, which is beneficial for nerve regeneration.	Schaakxs, et al. [46]
PHB	-	Chitosan	Implant	Co-precipitation	The implant supported osteochondral regeneration and could improve cartilage tissue regeneration.	Petrovova, et al. [47]
PHB	Hydroxyapatite and mesenchymal stem cells	Alginate hydrogel	Bioactive biopolymer/mineral/hydrogel scaffold	Salt leaching technique and 3D- printing	The scaffold induced the osteogenic differentiation of mesenchymal stem cells.	Volkov, et al. [48]
PHB	-	Bacterial cellulose	Bone grafts	Salt leaching	The scaffolds supported 3T3-L1 preadipocyte viability and proliferation without toxicity and showed promising biocompatibility.	Codreanu, et al. [49]
PHB and PHBV	-	Anionic sulfated polysaccharide κ-carrageenan (κ-CG)	Fiber	Electrospinning	κ-CG loaded PHBV fibers showed good bioactive and osteogenic properties.	Goonoo, et al. [50]
PHBHHx	Neural stem cells	-	Film	Solution casting	PHBHHx did not trigger reactive gliosis as well as survival and growth of the transplanted stem cells in a rat traumatic brain injury model	Wang, et al. [51]
PHBHHx	Recombinant BMP-2 proteins	-	Porous structured scaffold	Solvent casting-particulate leaching	The porous biocompatible scaffolds successfully formed a network of blood vessels and promoted bone regeneration in rabbit radius.	Liu, et al. [52]
PHBV	Tachyplesin I (Tac) peptide	-	Film	Solution casting	The surface functionalization of PHBHV with Tac improved antibacterial and fibroblast proliferation.	Xue, et al. [53]
PHBV	Cerium oxide nanoparticles	-	Membrane	Electrospinning	The cerium oxide nanoparticles loaded PHBV membranes enhanced cell proliferation, vascularization and promoted the healing of diabetic wounds.	Augustine, et al. [54]
PHBV	Insulin-producing cells	-	Nanofibers	Electrospinning	PHBV was found to increase the survival rate of insulin-producing cells. Insulin-producing cells in combination with PHBV is a promising cell-copolymer construct that could be used for pancreatic tissue engineering applications.	Abazari, et al. [55]
PHBV	Vascular endothelial growth factor (VEGF), basic fibroblast growth factor (bFGF) and stromal cell-derived factor 1α	Poly(caprolactone)	tissue-engineered vascular graft	Electrospinning	PHBV based graft showed high biocompatibility and calcification resistance as well as a moderate haemocompatibility but was prone to aneurysmatic dilation.	Antonova, et al. [56]
PHBV	Arg-Gly-Asp peptide	Poly(caprolactone)	Patches	Electrospinning	The PHBV based patches showed neointima formation and continuous endothelial lining on their surface.	Sevostianova, et al. [57]
PHBV	Vascular endothelial growth factor and platelet factor concentrate	Poly (vinyl alcohol) and elastin nanofiber	Fibrous scaffold	Electrospinning	The tri-layered scaffold was compatible to blood and promising for small diameter vascular grafting.	Deepthi, et al. [58]
PHBV	-	Polyethylene oxide	Nanofiber film	Electrospinning	When tested in a nerve rat model, the PHBV incorporated with polyethylene oxide promoted peripheral nerve regeneration.	Zhang, et al. [59]
PHBV	Quercetin	-	Fibrous scaffolds	Electrospinning	The scaffolds facilitated growth of chondrocytes and maintained chondrocyte phenotype and inhibited apoptosis and reduced oxidative stress of chondrocytes.	Chen, et al. [60]
PHBV	-	Aloe vera gel	Nanofibrous scaffold	electrospinning	The aloe vera gel-blended PHBV scaffold showed promising osteoinductive potential with complete degradation without harmful products.	Tahmasebi, et al. [61]
PHBV	Adenosine	-	Composite nanofiber	Electrospinning	The composite nanofiber showed good tissue biocompatibility and promoted bone regeneration capacity in vitro and in vivo.	Zhong, et al. [62]
PHBV	Epidermal growth factor	Gelatin-methacryloyl	Hydrogel patches	Electrospinning	The drug-loaded patches provided promising cellular response, angiogenesis and wound healing.	Augustine, et al. [63]
PHBV	Silver nanoparticles	High molecular weight keratin	Nanofibrous mat	Electrospinning	The nanofibrous mat demonstrated favourable mechanical and antibacterial properties with good biocompatibility, makes it suitable for wound healing.	Ye, et al. [64]
PHBV	-	-	Nanofibrous meshes or film	Electrospinning or solution casting	The electrospun nanofibrous meshes were better in mitigating excessive scar formation by regulating myofibroblast formation through downregulation of α-SMA and TGF-β1, and upregulation of TGF-β3 as compared to the solution-cast films.	Kim, et al. [65]
PHO	-	-	Patch	Electrospinning	The PHO patches were as good as collagen in cell viability, proliferation, and adhesion with enhanced cell adhesion and proliferation.	Bagdadi, et al. [66]

* [(P34HB), poly3-hydroxybutyrate-*co*-4-hydroxybutyrate; [PHBV)], poly(3-hydroxybutyrate-*co*-3-valerate); (PHBHHx), poly(3-hydroxybutyrate-*co*-3-hydroxyhexanoate).

## Data Availability

Not applicable.
